# Metabolite Profiling and Anti-Inflammatory Activities of *Fritillaria cirrhosa* D. Don Bulbs Derived from Tissue Culture

**DOI:** 10.3390/molecules30030623

**Published:** 2025-01-31

**Authors:** Yu Wang, Jiamin Liu, Enhao Zhang, Yixi Yang, Qiuxia Lu, Ziwei Zhu, Rui Li

**Affiliations:** 1 Natural Products Chem-Bio Innovation Center, Chengdu University, Chengdu 610106, China; wangyu123@cdu.edu.cn (Y.W.); liujiamin@cdu.edu.cn (J.L.); zhangenhao@cdu.edu.cn (E.Z.); yangyixi1011@cdu.edu.cn (Y.Y.); luqiuxia@cdu.edu.cn (Q.L.); 2 Engineering Research Center of Sichuan-Tibet Traditional Medicinal Plant, Chengdu University, Chengdu 610106, China; 3 School of Pharmacy, Chengdu University, Chengdu 610106, China; 4 School of Food and Biological Engineering, Chengdu University, Chengdu 610106, China; 5 Institute for Advanced Study, Chengdu University, Chengdu 610106, China

**Keywords:** *Fritillaria cirrhosa* D. Don, tissue culture, UHPLC-Q-TOF/MS, TRPV1, anti-inflammatory

## Abstract

*Fritillaria cirrhosa* D. Don (known as Chuan-Bei-Mu in Chinese) is a prominent medicinal plant utilized in traditional medicine for chronic respiratory ailments. It has garnered global acknowledgment because of its incorporation in many herbal preparations, resulting in a significant increase in demand and, consequently, leading to the decimation of wild populations. The study aimed to obtain regenerated plantlets of *F*. *cirrhosa* using in vitro propagation techniques and evaluate the accumulation of active metabolites and anti-inflammatory properties from in vitro and natural plant bulbs. UHPLC-Q-TOF/MS analysis identified 267 metabolites. Notably, 118 metabolites showed significantly different intensities between the wild bulbs (WBs) and in vitro tissue culture-regenerated bulbs (RBs). Higher edpetiline amounts were obtained from the RBs, and 14 steroid-related metabolites were elevated in RBs. Both RB and WB extracts had comparable anti-inflammatory abilities and significantly inhibited TNF-α-induced epithelial cell TSLP release. Subsequent mechanistic studies revealed that the efficacy of WB and RB extracts depended on the regulation of the TRPV1/NFAT pathway. These findings highlight the viability of in vitro regeneration and medicinal part replacement as sustainable alternatives to the existing detrimental overharvesting of wild Chuan-Bei-Mu populations.

## 1. Introduction

*Fritillaria cirrhosa* D. Don, commonly known as Chuan-Bei-Mu, is a perennial medicinal herb belonging to the family Liliaceae. The bulbs of *F. cirrhosa* are commonly used for the treatment of lung-related diseases, such as asthma, cough, lung cancer, and tuberculosis [1]. Notably, these medicinal uses have been documented in Divine Farmer’s Materia Medica (Shennong Bencao Jing). Modern pharmacological studies have confirmed that *F. cirrhosa* has anti-asthmatic, antioxidant, antitumor, and anti-inflammatory functions [2,3,4]. Using *F. cirrhosa* in medicine has resulted in an increasing trend of incorporating this plant as the primary component of various medications, such as the “Chuanbei Zhike Lu” decoction, “Ermu Ningsou Wan” pills, and “Xiao’ er Qingfei Zhike Pian” pills, among other medications [5]. The medical value of *F. cirrhosa* has drawn interest from companies and commercial exploiters. Notably, the collection of *F*. *cirrhosa* plants from the wild remains the primary source of raw materials, which has resulted in the over-exploitation of limited natural *F. cirrhosa* resources [6]. In addition, *F. cirrhosa* exhibits a comparatively protracted growth cycle and yields are quite low [7]. *F. cirrhosa* is currently considered a critically endangered species and is categorized as a Class II protected species in China’s “wild Official Species under Protection of the State according to Wild Medicine Material Protection Rules” (http://www.cites.org.cn/, accessed on 5 May 2024). Plant tissue culture shows great potential as an alternative approach for generating strong bioactive chemicals of medical importance to overcome these constraints [8].

Plant tissue culture technology provides a feasible strategy for improving the production of active compounds in medicinal plants [9], especially rare medicinal plants that are slow-growing and difficult to cultivate because of their specialized habitats. Moreover, many of the active compounds are characterized by a low content, complex structure, instability, and difficulty in chemical synthesis [10,11]. Plant tissue culture technology allows controlled growth and development of plant cells under manipulated environmental conditions to promote the production of required metabolites [12]. Studies postulate that hundreds of secondary metabolites can be produced by plant tissue culture technology [13]. In addition, some important secondary metabolic active substances, such as ginsenosides, paclitaxel, and artemisinin, can reach the industrial level after continuous optimization of in vitro culture conditions [11,13].

There are several species of Chuan-Bei-Mu in China. *F. cirrhosa* D. Don, locally known as Chuan-Bei-Mu, is among the most important species. Phytochemical studies of Chuan-Bei-Mu have demonstrated that these species contain a number of alkaloids responsible for their pharmacological activity [5]. The in vitro propagation of plants or the in vitro culture of plant organs (usually bulbs) or callus formation are optional methods for the production of these valuable alkaloids [14,15,16]. These methods can particularly lead to the production of higher imperialine content, which is typically used as a pharmacopoeia reference standard for quality assessments of Chuan-Bei-Mu [17]. Despite extensive knowledge of the phytochemicals of in vitro-derived propagules of *F. cirrhosa*, there has been a notable lack of research on the bioactivity of *F. cirrhosa* tissue culture products. It is, thus, necessary to establish a valid pharmacological activity evaluation method to control the quality of the in vitro-derived *F. cirrhosa* plants. Recently, an in vitro model of TNF-α-induced inflammation established in BEAS-2B cells demonstrated that the total alkaloids of *Fritillaria unibracteata* var. wabuensis bulbus could alleviate asthmatic inflammation by suppressing the TRPV1 pathway [18]. *F. cirrhosa* plants can potentially exhibit the same anti-inflammatory effect.

With the aid of advanced analytical tools, such as ultra-performance liquid chromatography-quadrupole-time-of-flight mass spectrometry (UHPLC-Q-TOF/MS), the chemical composition analyses have been successfully applied to Chuan-Bei-Mu [18]. This study, thus, aimed to evaluate the characteristics and amounts of alkaloid components (as biomarkers) in the regenerated bulbs of *F. cirrhosa* tissue culture using UHPLC-Q-TOF/MS analysis and to compare their anti-inflammatory activities with those of wild bulbs. The findings of this study provide a basis for future research into the potential medicinal and functional food uses of tissue culture *F. cirrhosa* plants.

## 2. Results and Discussion

### 2.1. Tissue Culture of F. cirrhosa

**Figure 1:**
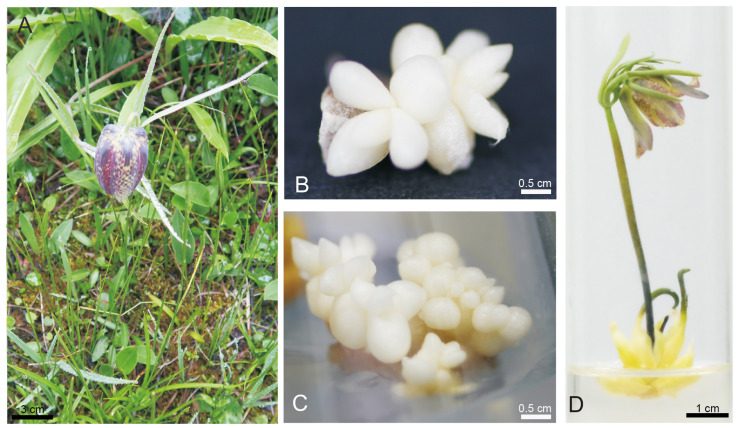
In vitro bulb regeneration of *F. cirrhosa*. (**A**) Wild plant. (**B**,**C**) Adventitious buds. (**D**) Regenerated bulblet.

In vitro plant cell and tissue culture has emerged as a sustainable regeneration technique for salvaging critically endangered plants and facilitating their restoration in natural habitats. It also offers a promising avenue for extracting bioactive compounds for use in the pharmaceutical industry. Standardized in vitro plantlet regeneration techniques have been developed for sustainable propagation of *Fritillaria* species using their bulb. Herein, the wild bulbs of *F. cirrhosa* were used as explants for in vitro plant regeneration (Figure 1A). Adventitious buds of *F. cirrhosa* can be generated in substantial quantities in a significantly reduced timeframe (seven to eight weeks). The adventitious buds exhibited a spherical and compact morphology (Figure 1B,C), leading to direct bulb development in the fourth month. The regenerated bulbs were produced in vitro through cytokinin/auxin induction. Notably, the differential gene expression between WBs and RBs and their metabolic composition and effects may be different. Moreover, bulblet micropropagation is more rapid and simpler than propagation via seeds and vegetatively by daughter bulbs, and RB may serve as a substitute for natural *F. cirrhosa.* Uniformly sized bulbs (bulb perimeter: 2.0 cm; plant height: 6.0 cm) (Figure 1D) of *F. cirrhosa* plantlets at their reproductive stage were, thus, analysed in detail to explore their application value for medicinal and food purposes. Of note, the selection of seed propagation for *Fritillaria* bulb production is of low application value because it takes five to six years for *Fritillaria* to develop from seed into commercially mature bulbs [19]. Herein, good-sized bulbs were obtained in four months using standardized and stable in vitro tissue culture for bulb propagation. Regenerated bulbs provide large amounts of medicinally active ingredients within a short time and are a source of material for the production of artificial *F. cirrhosa* seeds [20].

### 2.2. Ion Chromatography Analysis

**Figure 2:**
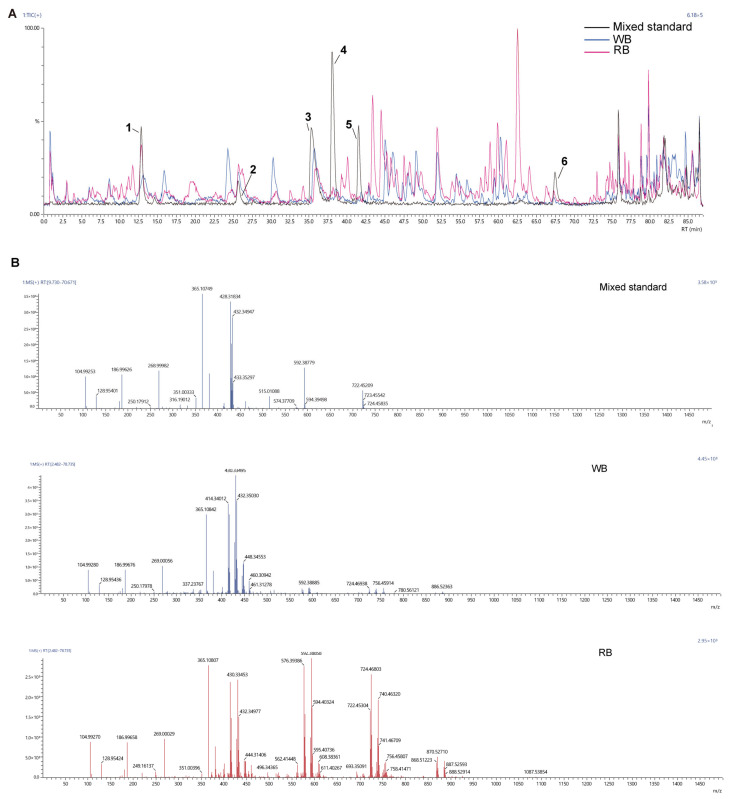
Identification of the chemical composition of WB and RB. (**A**) Total ion current (TIC) diagram of WB, RB and mixed standards. (**B**) The ion-averaged mass-to-nucleus ratio (*m/z*) of WB, RB and mixed standards.

It is essential to ascertain the phytochemical content of tissue culture plants before availing them to markets, farmers, or industrial users. The UPLC-Q-TOF/MS technique has been established and validated for the identification and quantification of *F. cirrhosa* alkaloids [18]. By scanning the precursor ions of the six compounds in the mixed standard and detecting the corresponding ion signals in the WB/RB, the results showed the presence of precursor ions in the WB/RB similar to those in the mixed standard, indicating the presence of these six compounds in the extract (Figure 2, Table 1). The mass spectrometry data (mzML format) were imported into MS-dial for analysis and pre-processing, such as peak extraction, peak alignment, etc., and the final metabolites in 267 were obtained by comparing them with the customized text library (Table S1). Edpetiline was the predominant alkaloid in the mixed standards and in the mass spectrometry analysis, Edpetiline from RBs showed a greater response in the total ion flow peak than in WBs. (Figure 2, Table 1). Previous studies using RNA-seq and metabolite expression profiling postulate that sipeimine accumulation may be positively correlated with gene expression in the biosynthetic pathway in callus, regenerated plantlets, and naturally grown bulbs of *Fritillaria roylei*. These reports suggest that callus culture can be used as a promising source for amplifying steroid alkaloid biosynthesis in genetic and metabolic engineering [15,21]. On this basis, researchers have continuously optimized the induction culture conditions, and successfully induced the regeneration bulbs of *F. cirrhosa* callus and detected the presence of some steroidal alkaloids [14,20,22]. Herein, regenerated bulbs of *F.cirrhosa* were also induced using tissue culture. An accumulation of steroidal biomass that was essentially comparable to that of wild bulbs was also detected, further demonstrating the potential for large-scale production of steroidal alkaloids from regenerated bulbs.

### 2.3. Chemotypic Differences in WB and RB Metabolites

**Figure 3:**
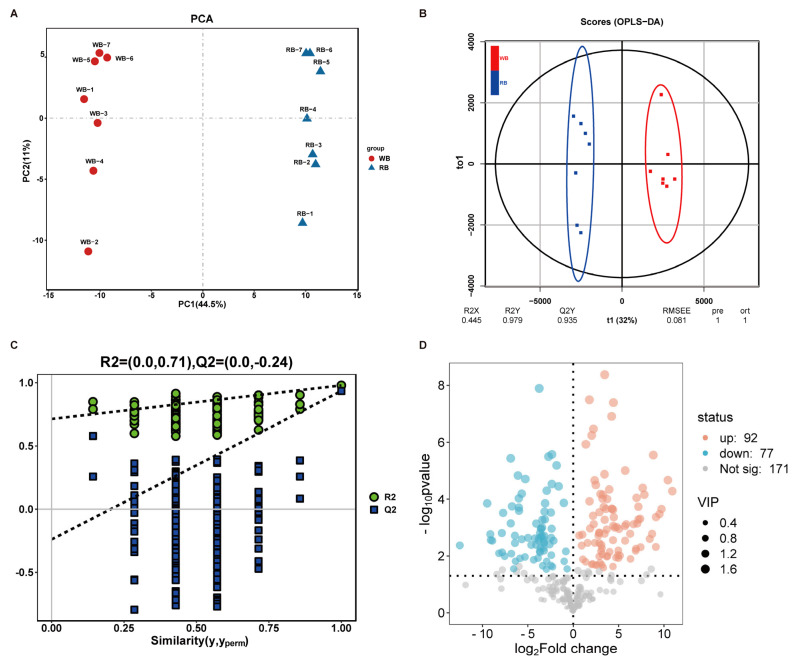
Chemotypic differences in WB and RB metabolites. (**A**) PCA score plots for WBs and RBs. (**B**) OPLS-DA score plots for WB and RB metabolites. (**C**) Plot of permutation test analysis for WB and RB metabolites. (**D**) Differential metabolite volcano plots for WBs vs. RBs.

PCA (Principal Component Analysis) and OPLS-DA (Orthogonal Partial Least-squares Discrimination Analysis) were employed to characterize the global metabolic differences between WB and RB metabolites. Figure 3A shows the PCA score plots of WB vs. RB metabolites. Principal component 1 (PC1) and PC2 accounted for 44.5% and 11% of the variation, respectively. The plots of the OPLS-DA score and permutation test demonstrated valid modelling and predictive abilities (Figure 3B,C). These results indicated that the metabolites accumulated in the WB vs. RB had a distinct separation. Notably, a total of 118 differentially accumulated metabolites (DAMs) were identified, including 61 upregulated and 57 downregulated, along with 149 metabolites that showed no significant changes between WBs and RBs (Figure 3D, Table S2). These metabolites were selected based on the following screening criteria: VIP (Variable Importance in Projection) value > 1, *p*-value < 0.05, and |log2(Fold Change)| ≥ 1. Steroidal alkaloids from *Fritillaria* species are the main bioactive compounds known for their diverse pharmacological effects [5]. Herein, 16 steroid differential metabolites (14 upregulated and 2 downregulated steroids in WBs vs. RBs) with significant changes selected (Table 2). Inferencing these findings with those of our prior transcriptome research, it appeared that the steroidal DAMs are potentially associated with the differential expression pattern of genes related to the steroid compound synthesis pathway in WBs vs. RBs [22]. Noteworthy, the accumulation of major steroidal alkaloid components did not change significantly in WBs vs. RBs; the accumulation in the RBs was comparable to that in the WBs (Figure 3D). In addition, the content of some other similar active steroidal compounds is higher in RB (Table 2). These compounds have various pharmacological properties, such as anti-inflammatory and anticancer [23,24], which enhance the medicinal function of *F. cirrhosa*-regenerated bulbs.

### 2.4. WB and RB Attenuate the Expression of Inflammatory Factors

TSLP is an “alarm factor” secreted by the skin’s mucosal barrier when it is irritated. Numerous studies postulate that TSLP acts as a master switch in the immune system, triggering and sustaining the course of some allergic diseases [25]. Intrinsic immune cells (macrophages, DC cells, and ILC2 cells) are further activated to release large amounts of pro-inflammatory factors, such as TNF, and activate humoral immunity when they receive TSLP signals. TNF-α can further stimulate the secretion of TSLP in epithelial cells. The two cellular exchanges form an inflammatory cycle to exacerbate the allergic reaction. Activation of this pathway is one of the key pathways driving disease development, especially in respiratory diseases such as asthma [26,27]. Intervention TSLP can be an effective anti-inflammatory strategy that can improve respiratory diseases, such as asthma. Kurihara and Numazaki et al. demonstrated that monoclonal antibodies, such as ASP7266 and Tezepelumab, alleviate inflammation and treat allergic asthma by inhibiting TSLP/TSLPR interactions [28]. Similarly, Mousa et al. demonstrated that Thyme oil relieves Ova-induced bronchial asthma by inhibiting TSLP [29].

**Figure 4:**
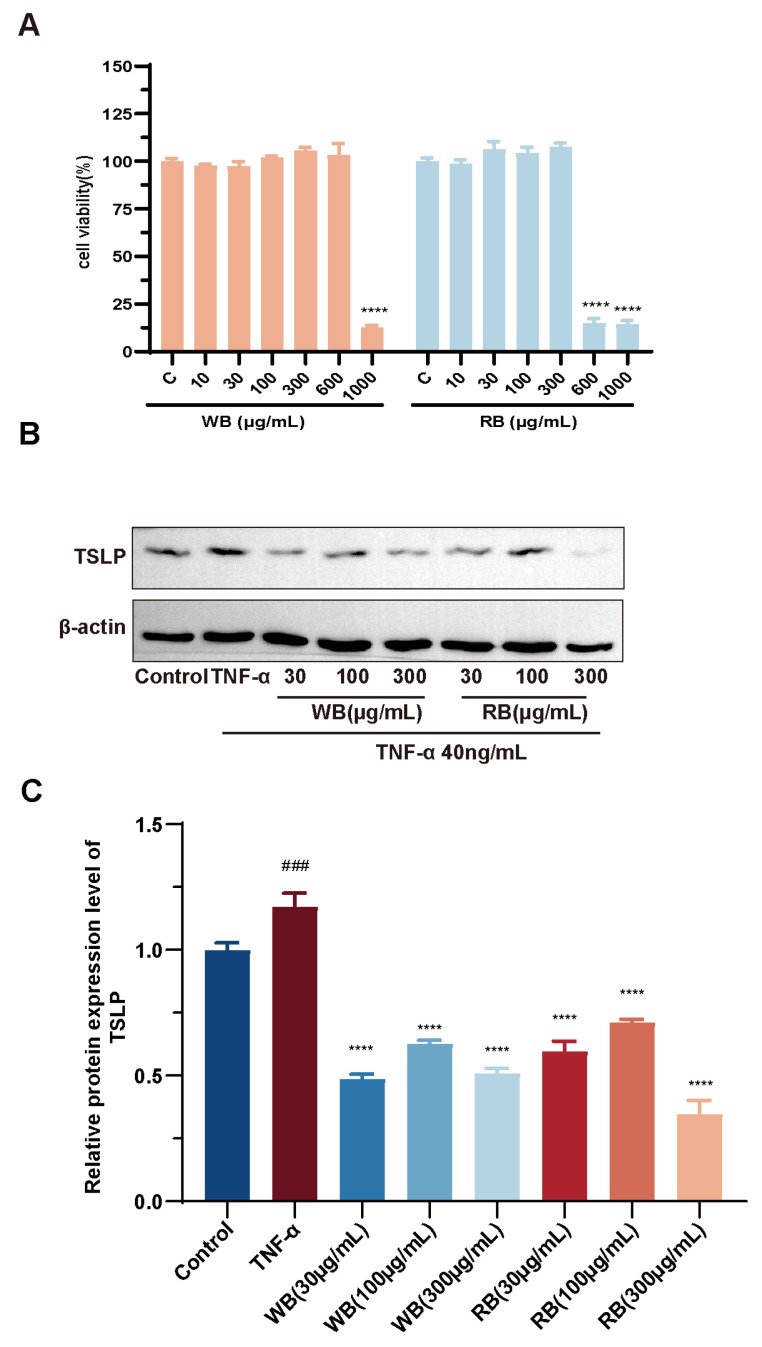
WB and RB attenuate the expression of inflammatory factors. (**A**) Cell viability was determined by CCK8 assay after treatment of Beas-2B cells with WB and RB (0–1000 μg/mL) for 24 h. (**B**) TSLP protein expression levels. (**C**) Quantitative analysis of TSLP protein levels. Data are expressed as mean ± SD (n = 3 per group; ### *p* < 0.001, compared with normal control, **** *p* < 0.0001 compared with the model group).

Steroid alkaloids are a diverse group of alkaloids isolated from *Liliaceae* and *Solanaceae* species and exhibit a wide range of biological activities [30,31]. Colchicine attenuates interstitial lung disease in a mouse model of experimental autoimmune myositis by inhibiting neutrophil extracellular trap formation [32]. Here, by performing cytotoxicity assays of WBs and RBs against Beas-2B, it is noteworthy that WBs and RBs were not toxic to the cells when the concentration was lower than 300 mg/mL (Figure 4A). Three concentrations, 30 μg/mL, 100 μg/mL, and 300 μg/mL, were thus used for the subsequent experiments. The TNF-α-inflammatory model in Beas-2B cells was adopted to explore the inflammatory effect of RBs and WBs. The WB results revealed that inflammatory mediators, such as TNF-α, stimulated the production and release of TSLP. Notably, both WBs and RBs effectively reduced the expression of TSLP. These findings preliminarily demonstrate the efficacy of WBs and RBs in inhibiting the activation of airway epithelial cells (Figure 4B,C).

### 2.5. Inhibition of TRPV1 Signaling Pathway by WB and RB Suppresses Inflammatory Response in Beas-2B Cells

The expression of TSLP is controlled by multiple transcription factors, of which NFAT is one of the modulators [32]. The transcriptional activity of NFAT is regulated by calmodulin; it translocates into the nucleus in response to intracellular Ca^2+^ [33,34,35]. The influx of Ca^2+^ is tightly controlled by TRPV family proteins, with TRPV1 playing a pivotal role. TRPV1 was originally identified in neurons, and recent studies have revealed significant expression of TRPV1 in the respiratory epithelial cells of asthma patients [36]. Further investigations have demonstrated that activating factors can stimulate TRPV1 activation, leading to an increased influx of Ca^2+^ and exacerbation of asthma symptoms. Inhibition of TRPV1 has been shown to alleviate airway inflammation [18]. Therefore, the regulation of the TRPV1/Ca^2+^/NFAT signaling pathway emerges as a crucial mechanism for alleviating asthma. *Fritillaria unibracteata* var. wabuensis extract has been shown to modulate the TRPV1/ Ca^2+^/NFAT pathway thereby inhibiting TSLP transcription [18]. Similarly, 20S-Ginsenoside Rh2 ameliorates airway inflammation by down-regulating TRPV1 expression [37]. Zhou et al. postulated that Houpo Mahuang Decoction reduces airway inflammation in asthmatic rats by inhibiting the activation and expression of TRPV1 and decreasing intracellular Ca^2+^ concentration [38].

**Figure 5:**
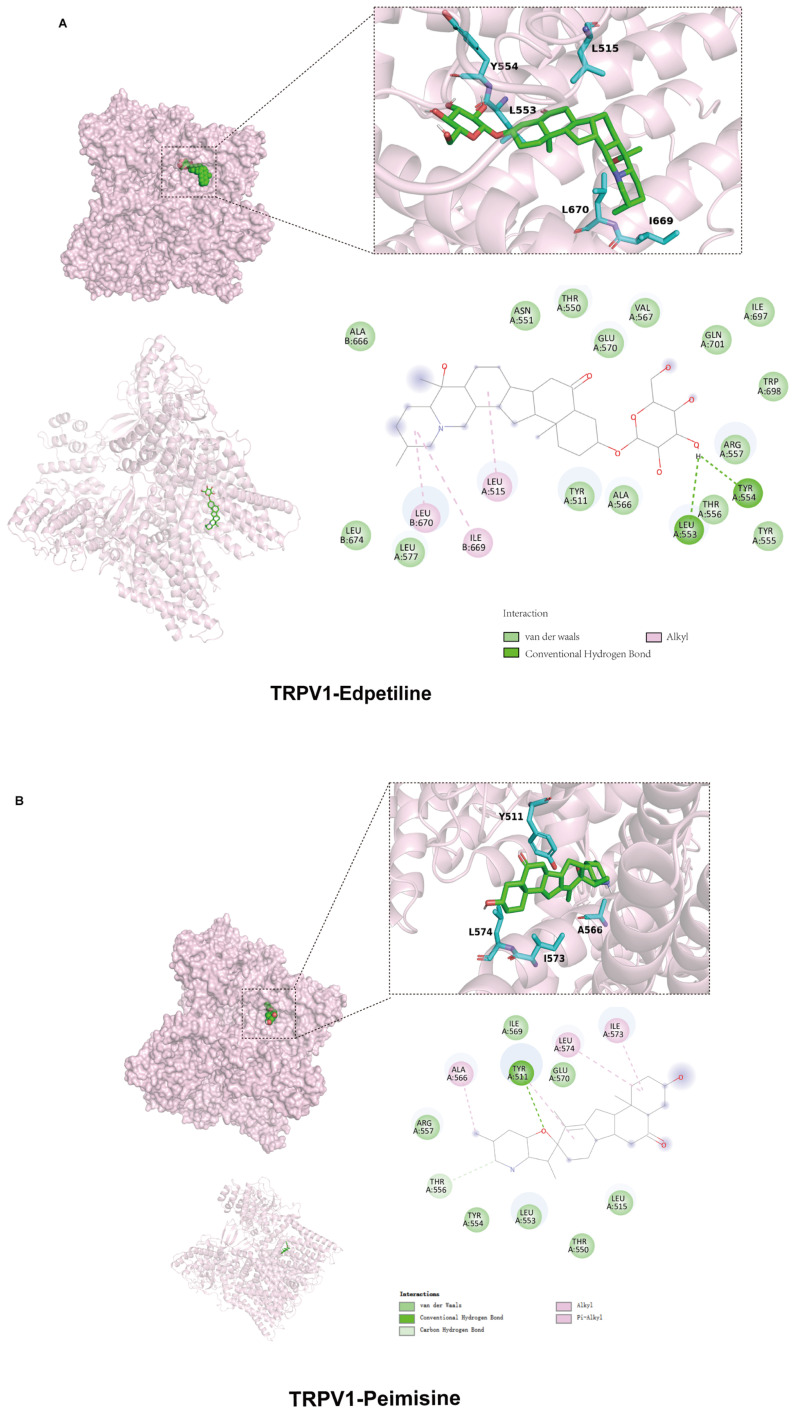
Molecular docking of edpetiline and peimisine with TRPV1. (**A**) Molecular-docking mode of edpetiline and TRPV1. (**B**) Molecular-docking mode of peimisine and TRPV1.

**Figure 6:**
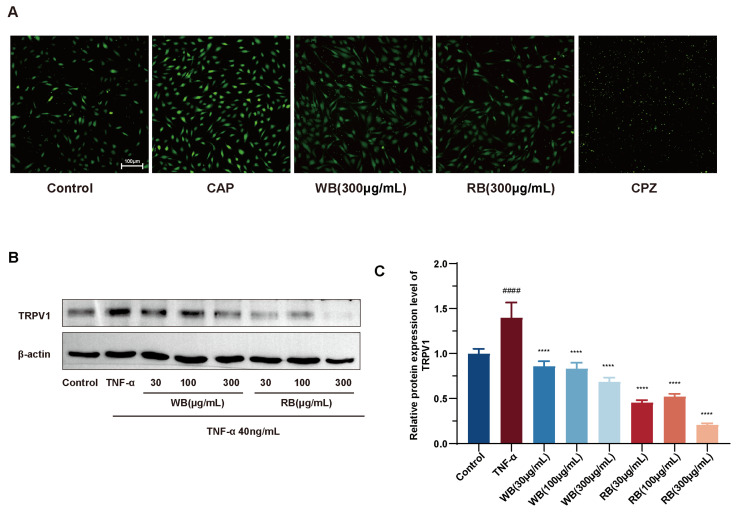
Inhibition of TRPV1 signaling pathway by WB and RB suppresses inflammatory response in Beas-2B cells. (**A**) WB and RB (300 μg/mL) and CPZ (20 μM) interfered with TNF-α (40 ng/mL) sensitized CAP (40 μm) to excite BEAS-2B cell fluorescence images. (**B**) TRPV1protein expression levels. (**C**) Quantitative analysis of TRPV1 protein levels. Data are expressed as mean ± SD (n = 3 per group; #### *p* < 0.0001 compared with normal control, **** *p* < 0.0001 compared with model group).

**Figure 7:**
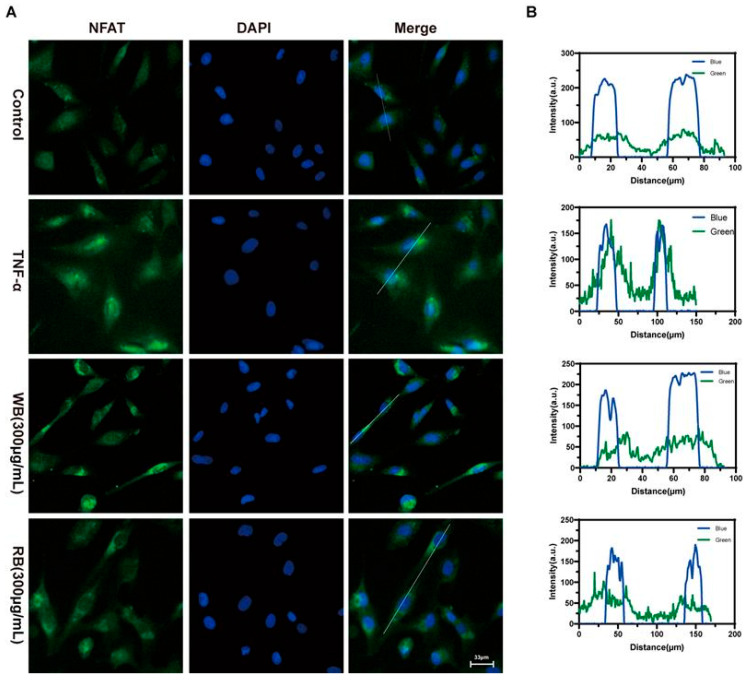
Inhibition of TRPV1 signaling pathway by WB and RB suppresses inflammatory response in Beas-2B cells. (**A**) Immunofluorescent labeling of dephosphorylated NFAT proteins in BeaS-2B cells and nuclei was labeled with DAPI. (**B**) Fluorescence intensity analysis of NFAT.

To further explore the mechanism between WBs and RBs and TRPV1. We selected representative components of WB and RB, edpetiline, and peimisine, for further molecular docking with TRPV1. The negative binding energy of molecular docking indicated that the docking was effective, and the smaller the binding energy value, the stronger the affinity of the small molecule compounds to the target protein. The results showed that edpetiline and peimisine interacted with TRPV1, and their binding free energies were both less than −5 kcal/mol. Hydrogen bonding was the main form of interaction. Edpetiline and TRPV1 formed hydrogen bonds at TYRA:554 and LEUA:553. Peimisine and TRPV1 formed a hydrogen bond at TYRA:511, which contributed to the formation of a hydrogen bond between the two compounds at TYRA:511, and the formation of a hydrogen bond at TYRA:511, which led to the formation of a hydrogen bond at TYRA:554. Hydrogen bonds are formed by 511, contributing to the stability of the binding. This result further validates that WB and RB can act on TRPV1 (Figure 5A,B). Herein, Western blotting assays confirmed that WB and RB inhibited TRPV1 expression (Figure 6B,C). The inhibitory impact of WB and RB on Ca^2+^ influx was further investigated utilizing flow-4 AM. Flow-4 AM generated fluorescence with an intensity that correlates with the intracellular Ca^2+^ concentration upon binding to intracellular Ca^2+^. TNF-α-sensitized Beas-2B cells showed enhanced fluo-4 AM fluorescence after 20 min of CAP stimulation, compared to cells treated with CAP alone. In contrast, treatment with WB, RB, and CPZ attenuated the sensitizing effect (Figure 6A). It is suggested that these stimuli increase the nuclear translocation of NFAT, as the nuclear level of dephosphorylated NFAT was elevated in Beas-2B cells treated with TNF-α (40 ng/mL) compared to untreated cells. However, both WBs and RBs reversed the nuclear translocation of NFAT with a decrease in nuclear fluorescence intensity (Figure 7A,B). In conclusion, mechanistic studies suggest that WBs and RBs can inhibit inflammation in epithelial cells via the TRPV1/Ca^2+^/NFAT pathway, thereby reducing TSLP release.

## 3. Materials and Methods

### 3.1. Chemicals

Methanol, chloroform, ammonia, ethanol and xylene were purchased from Chron Chemicals (Sichuan, China). The following chemicals were acquired from Supelco (Bellefonte, PA, USA): methanol, acetonitrile, and formic acid for use in UPLC-Q-TOF/MS. Standards such as edpetiline, khasianine, peiminine, peimisine, peimine, and sipeimine were acquired from MedChemExpress (Shanghai, China). The TNF-α protein (HZ1014), TSLP antibody (13778-1-AP) and TRPV1 antibody (66,983-1-1 g) were acquired from Proteintech (Hubei, China). The NFAT antibody (Sc-7294) was purchased from Santa cruz Biotechnology (Shanghai, China) and the β-actin (M1210-2) antibody was purchased from Huabio (Zhejiang, China). All chemicals used in the assays were of analytical grade.

### 3.2. Plant Material and In Vitro Tissue Culture Growth Conditions

Bulbs of wild *F. cirrhosa* were used as the source of explants for the establishment of bulblets culture, as previously described by Zhao et al. [22]. Briefly, the wild bulbs were first washed with running water (60 min), then successively rinsed with ethanol (70%, *v/v*; 30 s), HgCl_2_ (0.1%, *v*/*v*; 20 min), sterile water (5 times). The disinfected bulbs were inoculated on Murashige and Skoog (MS) medium (PH 5.8) supplemented with 30 g/L sucrose, 2 mg/L 6-BA (6-Benzylaminopurine) and 0.5 mg/L NAA (1-Naphthaleneacetic acid) (adventitious buds induction culture). After the adventitious buds formed, they were cultured on MS medium about 3 month until the bulbs matured (bulb perimeter: 2.0 cm; plant height: 6.0 cm), and then the bulb could be picked for the UHPLC-Q-TOF/MS and anti-inflammatory activities analysis. Cultures were maintained at 20 °C and 16 h light/8 h dark photoperiod.

### 3.3. Preparation of Plant Extracts

All collected bulbs were of uniform size, with an average circumference of 2.0 cm and an average height of 6.0 cm. WBs and RBs were dried separately at 37 °C to a constant weight and then ground into powder. Accurately weighed 2 g (to the nearest 0.1 mg) of the powder of WB/RB alkaloid, soaked in ammonia for 1 h at room temperature, then sonicated in chloroform–methanol (4:1) solution at low temperature for 2 h, filtered, and evaporated to near dryness by rotary evaporator, re-solubilized with water, and finally lyophilized at 5 Pa to obtain the alkaloids [39].

### 3.4. UHPLC-Q-TOF/MS Analysis of Alkaloids

A mixed standard solution of six alkaloids was prepared with an injection volume of 1 μL. Take 10 mg of WB/RB alkaloid and dissolve the alkaloid with 2 mL of mass spectrometry grade methanol, after all dissolved, dilute the solution to 1 mg/mL, pass through 0.22 pm organic phase filter membrane to 2 mL sample bottle to obtain the sample solution. The chemical composition of WB/RB total alkaloids (1 mg/mL) was analyzed by UHPLC-Q-TOF/MS (LC-MS 9030 Shimadzu, Japan). The chromatographic column of ACQUITY UPLC HSS T3 chromatographic column (2.1 × 100 mm, particle size: 1.8 μm; Waters, Georgija, USA) was used. The column temperature was 40 °C, the flow rate was 0.4 mL/min, and the mobile phase was 0.1% formic acid aqueous solution and acetonitrile (ACN). The gradient elution conditions were as follows: 0–1 min 15% ACN, 1–20 min 15% ACN, 20–60 min 15–30% ACN, 60–70 min 30% ACN, 70–80 min 30–95% ACN, 80–85 min 95% ACN, and 85–86 min 95–15% ACN. Using a data-dependent acquisition (DDA) mode range of 30–1000 *m/z*, an interface temperature of 300 °C, and an ion spray voltage of −3.50 kV, mass spectrometry was conducted in the cation mode.

### 3.5. UHPLC-Q-TOF/MS Data Preprocessing and Multivariate Analysis

The mass spectrometry data were exported in mzML format and analysed by MS-dial for filtering, peak extraction and peak alignment, and 267 metabolites were finally obtained by comparing with the custom text library. The identified metabolites were centred using SIMCA software (Version 14.1) to establish the principal component analysis (PCA) model and the orthogonal partial least squares-discriminant analysis (OPLS-DA) model. Differences were screened by variable importance in the projection (VIP) values, *p* values, and log2(Fold Change). Statistically significant differences in metabolite molecules were observed when VIP ≥ 1, *p* < 0.05, and |log2(Fold Change) | ≥ 1.

### 3.6. Cell Culture

WB and RB total alkaloids powder 90 mg, respectively, was dissolved with a small amount of Dimethyl Sulfoxide (DMSO), the content of which should not be more than 2/1000 of the concentration used, and subsequently partitioned and stored in the refrigerator at −80 °C, and diluted with culture medium for the subsequent cell experiments. Beas-2B cells used in the experiments were obtained from the Cell Bank of the Chinese Academy of Sciences (Shanghai, China). The cells were cultured in Dulbecco’s modified Eagle medium (DMEM, Wisent, ST-Bruno, Quebec, Canada) containing 10% fetal bovine serum (SAN Bruno, Quebec, Canada), 100 mg/mL streptomycin, and 100 U/mL penicillin. The cultures were maintained at 5% CO_2_ and 37 °C and were subsequently used to detect cell viability, immunofluorescence and for the Western blot assay.

### 3.7. Detection of Cell Viability with CCK8 (Cell Counting Kit-8 Assay) Kit

Beas-2B cells (8 × 10^4^) were plated in 96-well plates and incubated overnight. WB and RB (30–300 μg/mL) were then added separately and incubated for 24 h. The drug-containing medium was subsequently replaced with 90 μL of DMEM and 10 μL of CCK8 solution per well in accordance with the instructions of the CCK8 kit (C0037, Beyotime, Chongqing, China). The cultures were incubated at 37 °C and 5% CO_2_ for 40 min, followed by optical density (OD) measurement at 450 nm using a microplate meter.

### 3.8. Molecular Docking

The 3D crystal structures of the target proteins were downloaded from the PDB protein database (https://www.rcsb.org/, accessed on 20 October 2024). Autodock Tools-1.5.7 was used to prepare the structure file for docking where the binding centre coordinates of the protein were (center_x = 145.258, center_y = 112.918, center_z = 142.001). Molecular docking experiments were performed using autodockvina 1.5.6 software. The maximum limit for searching conformations was set to 100, and genetic algorithms were used for conformational sampling and scoring, and the optimal structures were selected based on the docking scores for conformational ranking and conformational reasonableness. Finally, the binding results were visualized by PyMOL 2.4.1 software.

### 3.9. Immunofluorescence Assay for Detection of NFAT Entry into the Nucleus of Cells

Cells were treated with the same culture as described in the previous section, and the original culture medium was discarded. The cells were then fixed using pre-cooled 4% paraformaldehyde for 15 min, permeabilized with 0.1% TritonX-100 for 15 min at room temperature, and then closed with 5% bovine serum albumin for 1 h. The cells were then incubated at 4 °C overnight with 1% bovine serum albumin diluted with NFAT antibody in the ratio of 1:100. The cells were subsequently incubated with secondary antibody for 1 h at room temperature under light-protected conditions, after which an antifluorescent burst containing 4′,6-diethylenediamino-2′-phenylindole was added and the cell mixture incubated for two minutes. The cellular images were subsequently acquired with a Nikon Eclipse Ni-U microscope (Tokyo, Japan).

### 3.10. Ca^2+^ Imaging

Beas-2B cells were first treated with WB and RB (300 μg/mL) and CPZ (capsazepine, 20 μM) for 24 h, as described in the earlier section, to explore the effects of WB, RB, and CPZ on TNF-α (40 ng/mL)-induced sensitization of TRPV1 channels. The cells were subsequently washed with PBS and treated in DMEM supplemented with 40 μM CAP (capsaicin) for 20 min. The Beas-2B cells underwent serum starvation for 24 h prior to imaging to investigate the inhibitory effects of the medications on cap-induced suppression of cellular Ca^2+^ influx. 40 μM and 10 μM CAP were used to activate Beas-2B cells for a duration of 20 min. In the group that received drugs, CAP stimulation was given at the same time as each drug. Following that, the cells were subjected to a 30 min incubation with Fluo-4 AM (S1060, Beyotime) for 30 min, washed with PBS, and subjected to Ca^2+^ endocytosis detection using a 516 nm fluorescence excitation. Cell images were subsequently captured using an inverted microscope.

### 3.11. Western Blotting

Beas-2B cells were seeded in 24-well plates at a ratio of 8 × 10^4^ cells/well and treated with TNF-α (40 ng/mL) and WB and RB (30, 100, 300 μg/mL) for 24 h. The total proteins in the Beas-2B cells were subsequently extracted using cell lysates containing RIPA 150 buffer, following the kit (R0050, Solarbio, Beijing, China) instructions. The protein samples were added to a 10% sodium dodecyl sulfate protein gel for electrophoresis and then transferred to a polyvinylidene fluoride membrane. The membranes were enclosed with 5% nonfat milk for 1 h, followed by overnight incubation at 4 °C in 1% bovine serum albumin containing TRPV1 (rabbit, 1:1000), TSLP (mouse, 1:1000), and β-actin (mouse, 1:10,000). The chemiluminescence signals were detected using the Extremely Sensitive ECL Chemiluminescence Kit (Oriscience, Chengdu, China) after incubation with secondary antibodies. The intensity of the protein bands was measured using MyImage software (iBright CL1500 Thermo Fisher Science, Waltham, MA, USA).

## 4. Conclusions

This article presents a comparative study of the phytochemical signature and anti-inflammatory activities of *F. cirrhosa* wild bulbs and in vitro-regenerated bulbs. UPLC-Q-TOF/MS data showed a strong similarity in bioactive medicinal compounds between WBs and RBs. Notably, edpetiline accumulation could be increased in vitro. This study validates the practical application of the in vitro propagation of Chuan Beimu in phytochemical substitution research. Both WBs and RBs possess similar anti-inflammatory properties and may substantially suppress TNF-A-induced TSLP production from epithelial cells. Nonetheless, mechanistic investigations indicated that their effectiveness relies on the modulation of the TRPV1/NAFT pathway. The findings of this study demonstrate the potential of employing in vitro culture techniques to produce the required bioactive metabolites in *F. cirrhosa* rather than relying on wild plants for pharmaceutical applications.

## Figures and Tables

**Table 1:** Mass spectral information of WB and RB (1 mg/mL, 1 μL) and mixed standards.

NO.	Target Name	Target Formula	RT ^1^ in Mixed Standard	RT ^1^ in the WB	RT ^1^ in the RB	*m/z* in Mixed Standard	*m/z* in WB	*m/z* in RB	Response ^2^ in Mixed Standard	Response ^2^ in the WB	Response ^2^ in the RB
**1**	Edpetiline	C_33_H_53_NO_8_	12.9874	12.6933	12.5427	592.38779	592.38885	592.38885	1,170,550	527,306	992,544
**2**	Sipeimine	C_27_H_43_NO_3_	26.0487	25.9012	25.9023	430.33157	430.33495	430.33453	502,110	757,323	868,954
**3**	Peimisine	C_27_H_41_NO_3_	36.0988	36.0113	36.0851	428.31834	428.31674	428.31805	2,208,535	1,024,700	987,868
**4**	Peimine	C_27_H_45_NO_3_	38.0533	38.3700	37.9571	432.34722	432.35030	432.34977	1,212,132	79,997	61,144
**5**	Peiminine	C_27_H_43_NO_3_	41.6200	41.4829	41.7672	430.33157	430.33495	430.33453	1,492,489	100,065	76,702
**6**	Khasianine	C_39_H_63_NO_11_	64.4504	67.4504	67.45404	722.45209	722.45195	722.45304	587,896	84,747	247,102

^1^ RT: Retention time, ^2^ Response: Total Ion Chromatogram (TIC).

**Table 2:** List of steroid differential metabolites in WBs vs. RBs.

Compound Name	Formula	Retention Time (min)	Fold Change	log2FC ^1^	*p*-Value	VIP ^2^	Type
Dehydroevodiamine	C_19_H_15_N_3_O	83.46	0.0018	−9.08	0.0027526	1.24	down
Veratramine	C_27_H_39_NO_2_	75.473	120.2589	6.91	0.0055646	1.19	up
Solasodine	C_27_H_43_NO_2_	51.608	52.7098	5.72	9.82 × 10^−6^	1.52	up
Solanidine base + O-Hex-dHex	C_39_H_63_NO_10_	61.293	52.3457	5.71	0.0029032	1.25	up
α-solanine	C_45_H_73_NO_15_	58.126	51.6251	5.69	0.0008903	1.33	up
Edpetinosine	C_33_H_55_NO_7_	49.52	32.0000	5	0.011857	1.1	up
Demissidine	C_27_H_45_NO	75.732	30.4844	4.93	0.0092492	1.13	up
Cycloposine	C_33_H_51_NO_7_	75.409	22.1618	4.47	0.0004324	1.36	up
Edpetiline	C_33_H_53_NO_8_	12.789	13.6422	3.77	0.0100805	1.12	up
Cyclopamine	C_27_H_41_NO_2_	79.683	13.2691	3.73	9.85 × 10^−5^	1.45	up
β--Sitosterol	C_29_H_50_O	85.805	12.9063	3.69	0.0011713	1.31	up
Tomatidine	C_27_H_45_NO_2_	47.999	0.0921	−3.44	0.0019842	1.28	down
Diosgenin	C_27_H_42_O_3_	80.086	9.7136	3.28	6.99 × 10^−5^	1.47	up
Sarsasapogenin	C_27_H_44_O_3_	79.977	5.1337	2.36	2.19 × 10^−5^	1.5	up
14-hydroxysprengerinin C	C_44_H_70_O_17_	74.336	4.5315	2.18	0.000384	1.36	up
Timosaponin A1	C_33_H_54_O_8_	75.292	2.9897	1.58	0.0034805	1.22	up

^1^ log2FC: log2 fold change, absolute values greater than 1 were considered significant differences in expression, ^2^ VIP: Variable Importance in Projection.

## Data Availability

The original contributions presented in the study are included in the article, further inquiries can be directed to the corresponding authors.

## Supplementary Materials

The following supporting information can be downloaded at: https://www.mdpi.com/article/10.3390/molecules30030623/s1, Table S1. The information of all identified metabolites between WB and RB and Table S2. The list of metabolites differentially expressed between WB vs RB.

## Abbreviations

The following abbreviations are used in this manuscript:

TSLP	Thymic Stromal Lymphopoietin
NFAT	Nuclear Factors of Activated T-cells
TRPV1	Transient Receptor Potential Vanilloid 1
TNF-α	Tumor Necrosis Factor-α
BEAS-2B	Bronchial Epithelium transformed with Ad12-SV402B
PCA	Principal Component Analysis
OPLS-DA	Orthogonal Partial Least Squares Discriminant Analysis
CAP	Capsaicin
CPZ	Capsazepine
6-BA	6-Benzylaminopurine
NAA	Naphthaleneacetic Acid
CAN	Acetonitrile
DMEM	Dulbecco’s modified Eagle medium
CCK8	Cell counting kit-8 assay
DMSO	Dimethyl Sulfoxide
